# Supplementary material for: Surrogate Biomarker Prediction from Whole-Slide Images for Evaluating Overall Survival in Lung Adenocarcinoma
Source: Diagnostics (Basel). 2024 Feb 20;14(5):462. doi: 10.3390/diagnostics14050462 (PMC10930763; doi:10.3390/diagnostics14050462)
Supplement: Supplementary file 1 [file diagnostics-14-00462-s001.zip › Supplementary_File_S3_Supplementary_Figures.pdf]

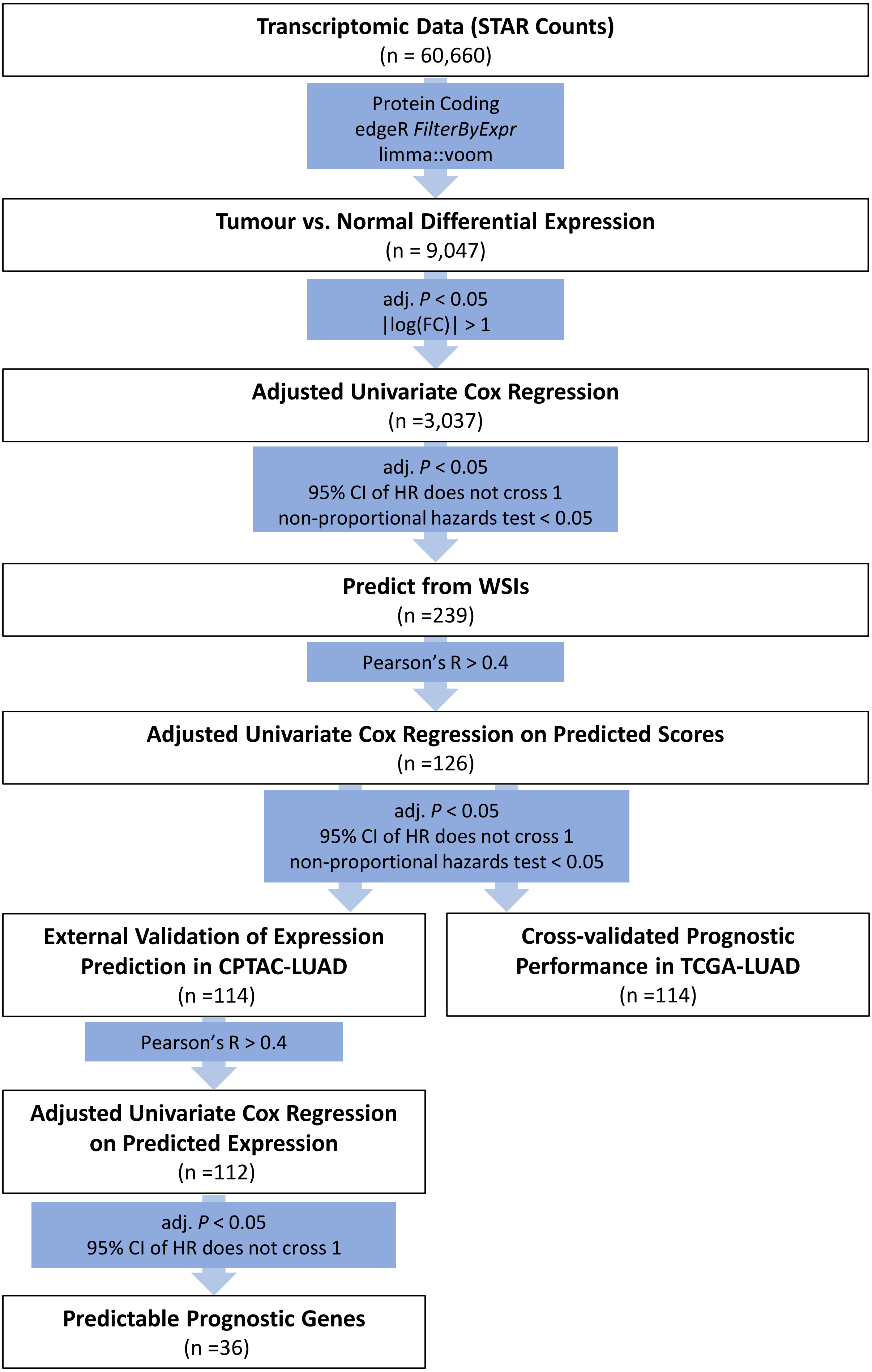

Suppl. Fig. 1: Study design flow chart.

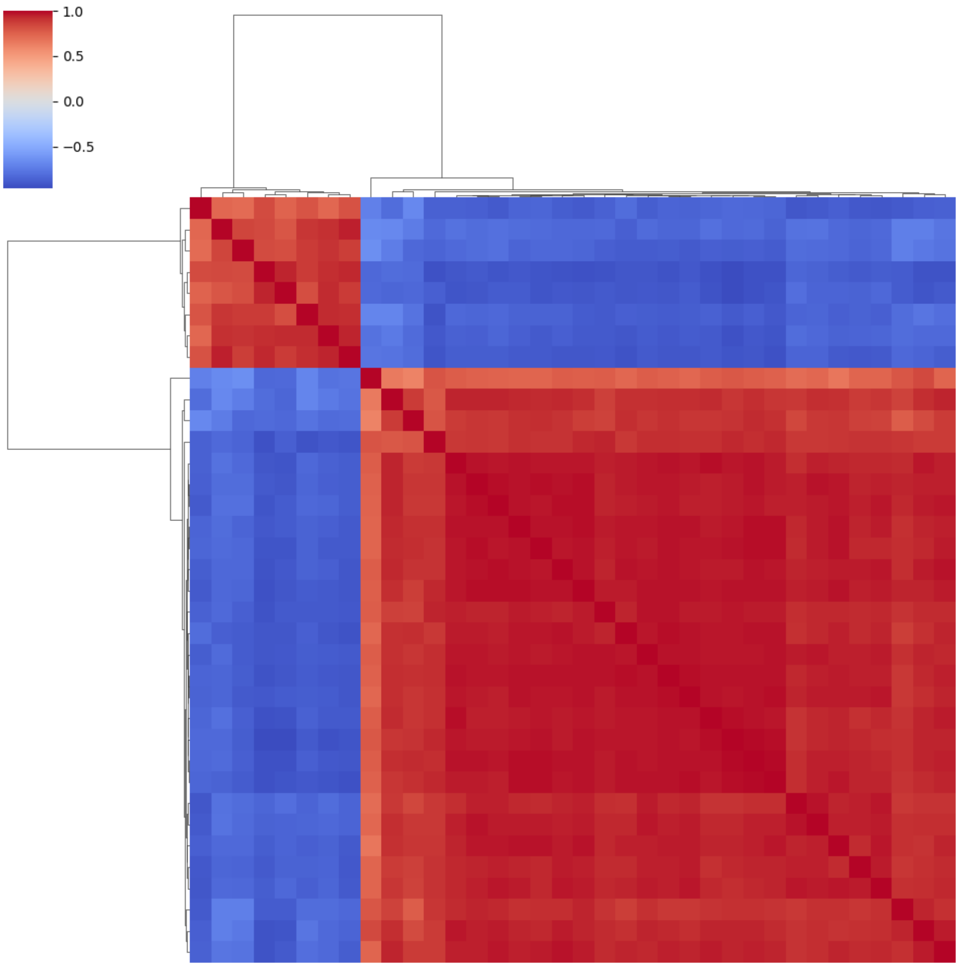

Suppl. Fig. 2: Clustergram of the predicted expression of the 36 genes that were found to have predicted expression that was prognostic of OS.

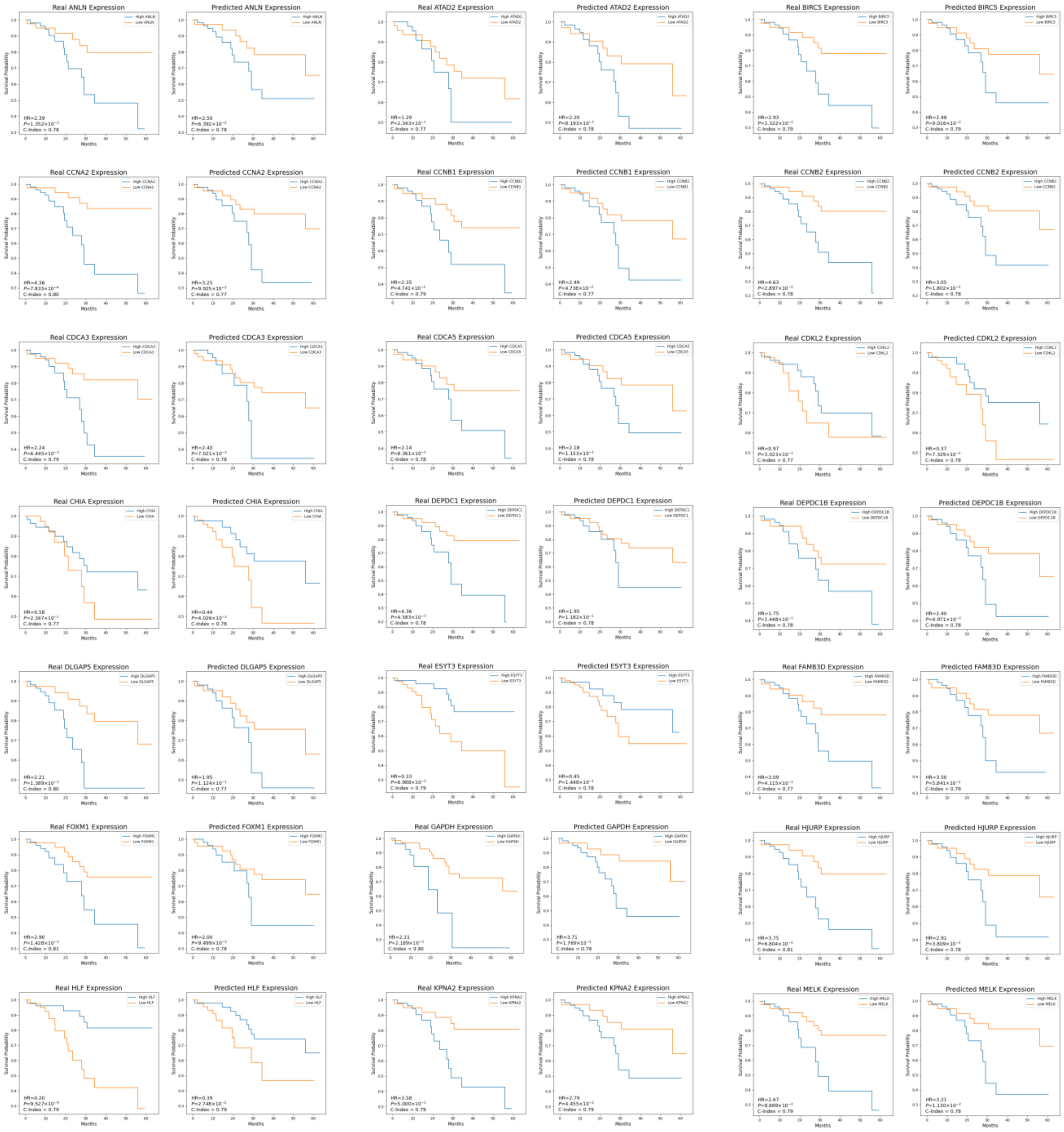

Suppl. Fig. 3: Kaplan-Meier plots of risk groups as determined using KMeans clustering with *k* set to 2 for real and predicted gene expression. Plots are shown for each of the 36 genes for which gene expression predicted from WSIs in the CPTAC-LUAD dataset was found to be prognostic of overall survival. Hazard ratios (HR) were determined by setting risk group as a covariate in Cox regression adjusting for age, stage, and sex. Corresponding concordance indices (C-index) are also shown along with log-rank *P*-values.

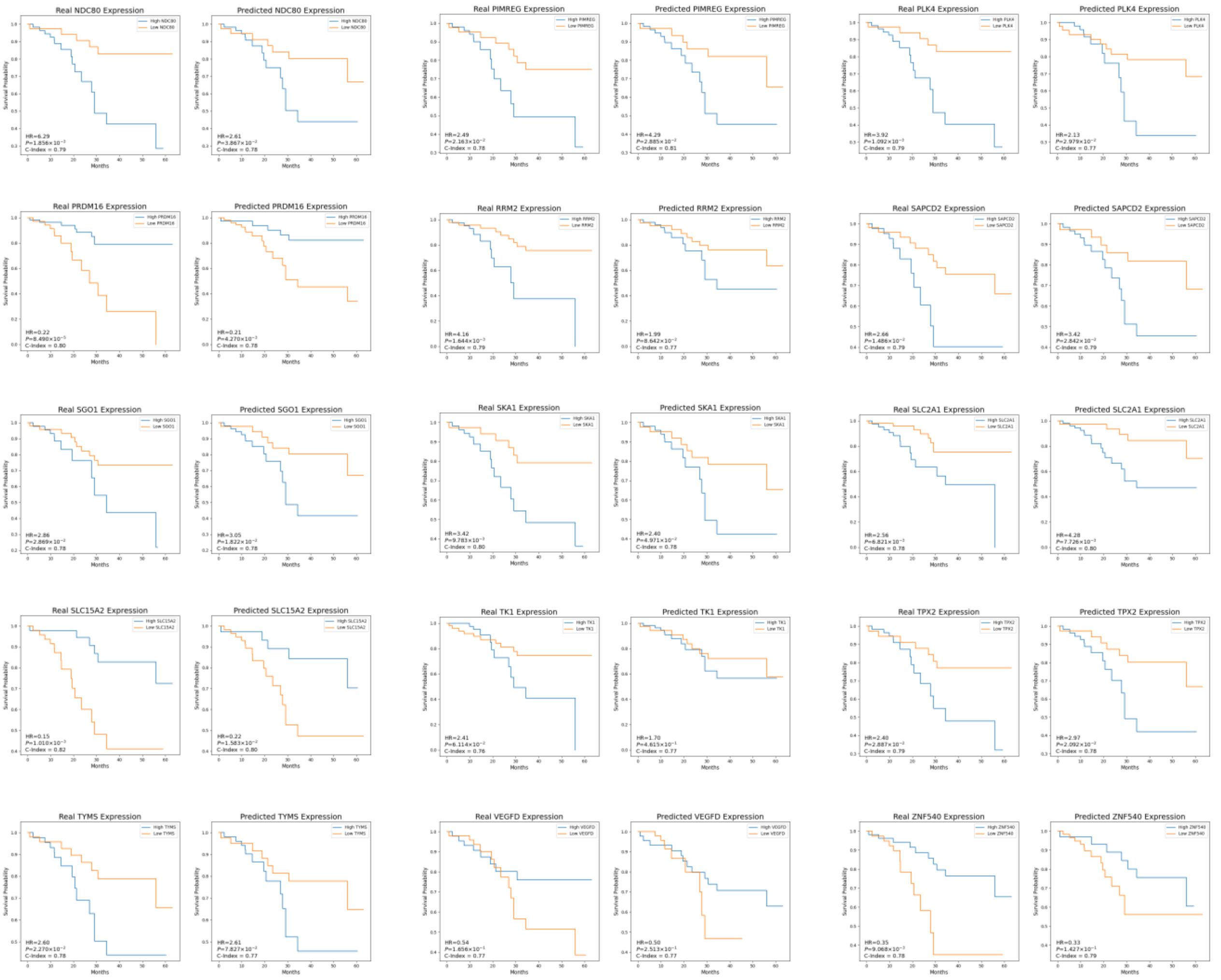

Suppl. Fig. 3 contd.
